# Stability and pH-Dependent Mechanism of Astaxanthin-Loaded Nanoemulsions Stabilized by Almond Protein Isolate

**DOI:** 10.3390/foods13244067

**Published:** 2024-12-17

**Authors:** Qingrui Yang, Wenhui Qi, Yutong Shao, Xu Zhang, Fengyang Wu, Zhisheng Zhang

**Affiliations:** 1 College of Food Science and Technology, Hebei Agricultural University, Lekai South Avenue, Baoding 071000, China; 17798136859@163.com (Q.Y.); wenhui406@yeah.net (W.Q.); 15176841005@163.com (Y.S.); 13703335994@163.com (X.Z.); fengyangwu2015@163.com (F.W.); 2 Market Supervision Administration Bureau of Luancheng District, No. 7 Xinyuan Road, Luancheng Town, Shijiazhuang 051430, China

**Keywords:** Pickering emulsion, almond protein isolate, astaxanthin, pH, interface distribution, stability

## Abstract

Pickering emulsions (PEs) of natural plant proteins enriched in fat-soluble components are gaining consumer interest for healthier and sustainable products. The aim of this study is to prepare PEs for stabilizing almond protein isolated (API) particles loaded with astaxanthin using ultrasound technology. The loose structure of the API at pH levels of 3 and 12, with contact angles of 68.92° and 72.56°, respectively, facilitated its transfer from the aqueous to the oil phase. The adsorption of the API at the oil–water interface was 71.56% and 74.69% at pH levels of 3 and 12, respectively, which was significantly higher than that of the emulsions at other pH levels (5, 7, and 9). After 14 days of storage at 4 °C, PEs at pH levels of 3 and 12 did not undergo phase separation, with small and homogeneous droplets. CLSM revealed a monolayer arrangement of the API at the oil–water interface. These results indicate that PE is more stable at pH levels of 3 and 12 than at other pH levels (5, 7, and 9). In addition, the stabilized astaxanthin PE showed the largest astaxanthin encapsulation (91.43%) at a pH of 3. The emulsions had significantly lower a* values and higher L* values at a pH of 3 compared to a pH of 12, indicating better astaxanthin stability in the PEs. These results will help to expand the application of API-PE loaded with astaxanthin at different pH values.

## 1. Introduction

Pickering emulsions (PEs) are formed by mixing immiscible liquids, such as oil and water. After high-intensity mechanical shearing, amphiphilic macromolecules (including protein macromolecules) or inorganic particulate surfactants are used to stabilize the dispersed phase within a continuous phase to form water-in-oil or oil-in-water emulsions [1,2,3]. PEs stabilized by solid particles exhibit higher stability and lower toxicity and irritation than surfactant-stabilized emulsions because of their specific resistance to agglomeration and Ostwald ripening [4]. The use of conventional emulsions with surfactants is becoming more restricted, considering the demand for naturalness, non-toxicity, biocompatibility, and high ecological acceptability [5,6]. The use of most inorganic nanoparticles (silica and clay) as emulsion stabilizers is limited because of their poor biocompatibility, biodegradability, and stability [7]. In contrast, natural plant protein particles are amphiphilic and used as emulsifiers with strong adhesion properties that can be stably suspended and dispersed in emulsions, forming a protein adsorbent layer at the oil–water interface to prevent separation and fusion. They also have low toxicity, making them safer and more eco-friendly than chemically synthesized emulsifiers, without posing health or environmental hazards [8,9]. Natural plant protein nanoparticles are of interest because they can be used to produce sustainable and clean food emulsions.

However, there are some limitations and challenges to using plant protein particles as emulsifiers. The emulsification of proteins is susceptible to external environmental influences such as temperature, pH, and salt ions, which affect the stability of PEs. Almeida et al. investigated amygdalin microgels produced by heat treatment, cross-linking with transglutaminase, or calcium ions at pH levels of 3 and 7 and explored their rheological properties [10]. Ghaedi et al. investigated the physical and oxidative stability of oil-in-water emulsions stabilized by bitter almond gum–soybean isolate protein couplings (1:1, 1:3, and 3:1) [11]. Huang et al. investigated the formation of stabilized PEs at a pH of 12 and enhanced oxidative stability of the emulsions using soy protein and carboxymethyl chitosan copolymers [12]. In related studies, most of the researchers aimed to prepare protein composite particles and protein–polysaccharide composite particles crosslinked [13], complexed [14,15], and glycosylated [16] to stabilize PEs. Single unmodified plant proteins have not been extensively studied as stabilizers of PEs. Therefore, identifying plant proteins with a wide pH range and low cost is challenging.

Plant-based proteins have emerged as potential sustainable emulsifiers. However, their applicability in stabilizing PEs, particularly with unmodified proteins, remains underexplored. Almond protein, containing the 18 essential amino acids required by the human body, is a good plant-based protein fortifier and an excellent raw material for biocompatible food-grade Pickering stabilizers [17]. Zhu et al. used ultrasound to improve the physical stability of oil-in-water emulsions stabilized with amygdalin [18]. The results of previous studies showed the preparation of API-stabilized PEs through ultrasonication and showed that APIs could act as emulsifying particles to stabilize PEs. However, investigations of the adsorption behavior of the API at the oil–water interface are scarce, and the difference in physical properties of PE prepared from API particles under acidic, neutral, and alkaline conditions is rarely discussed. Considering the recent demand for plant-based protein particles, it is necessary to describe the abilities of such PEs at various pH values to understand their responsiveness to environmental conditions during processing.

Astaxanthin (AST) is a fat-soluble carotenoid with many potential health benefits. However, its poor stability and pH sensitivity hinder its use as a functional food ingredient. Various strategies, including emulsion [19], liposome [20], and microencapsulation [21,22], have been developed to improve its stability and bioavailability in foods. In emulsions, dissolving AST in the oil phase with a large surface area can improve its dissolution and absorption [23]. Oil-in-water emulsions can improve the stability and bioavailability of stored carotenoids [24]. Zhang et al. prepared an oil-in-water PE stabilized by colloidal particles of konjac glucomannan/wheat glycolysin. The results showed that AST bioavailability reached 22.37 ± 0.62% when the mass ratio of wheat glycolysin to konjac glucomannan was 1:1, which is 4.5-fold higher than that of AST in pure oil [25]. Tang et al. studied AST-loaded buckwheat protein PEs and assessed their bioavailability in in vitro digestion experiments [26]. Gao et al. used ovalbumin (OVA) and gum arabic (GA) to prepare OVA-GA couplings and OVA-GA mixtures and then prepared AST-loaded oleogel-based nanoemulsions to improve the lipolysis and bioaccessibility of AST [27]. However, studies on the stabilization mechanism of AST in PE, particularly under different pH conditions, are lacking. The innovative application of API-PE for AST delivery addresses a crucial gap in stabilizing fat-soluble bioactives, paving the way for more effective formulations in food, nutraceuticals, and pharmaceuticals.

This study aimed to investigate the structural changes in almond protein isolates (APIs) at different pH values using sodium dodecyl sulfate-polyacrylamide gel electrophoresis (SDS-PAGE) and Fourier-transform infrared spectroscopy (FT-IR) and discuss their emulsification properties. Furthermore, the adsorption behavior of APIs at the oil–water interface at different pH values was evaluated using confocal laser scanning microscopy (CLSM), the dynamic scattering technique, and rheology. In addition, the protective effect of API-PE on AST was investigated, and the stabilization mechanism of AST in PE was revealed. This study supports the use of AST-loaded API-PEs in the food industry.

## 2. Materials and Methods

### 2.1. Materials

Sweet almond meal was generously provided by Zhangjiakou Yong Chang Yuan Kernel Food Co., Ltd. (Zhangjiakou, China). Soybean oil was purchased from the Huiyou Supermarket in Baoding, China. The Nile Red and Nile Blue reagents were purchased from Sigma-Aldrich (St. Louis, MO, USA). All other chemical reagents were purchased from Sinopharm Chemical Reagent Co., Ltd. (Shanghai, China). Unless otherwise indicated, all experiments were conducted using deionized water (DI water).

### 2.2. Preparation of APIs

APIs were extracted using the method described by Sze-Tao with some modifications [28]. Firstly, the almond meal was crushed, sieved (100 mesh), and degreased. The defatted almond powder was dispersed in DI water at a ratio of 1:20 (g/g), and its pH value was adjusted to 9.0 with 2.0 mol/L sodium hydroxide (NaOH). The defatted almond powder dispersion was stirred for 1.0 h at 25 °C and then centrifuged at 8000× *g* for 10 min at 4 °C. The sediment was dispersed in DI water again, and the alkali solution and centrifugation steps above were repeated. The two-part supernatant, after two centrifugations, was collected and merged. The pH of the collected supernatant was adjusted to a pH of 4.5 with 1.0 mol/L hydrochloric acid (HCL), and the slurry was stirred for 2.0 h at room temperature to obtain the crude protein suspension. The obtained crude protein suspension was left at room temperature for 0.5 h, and the supernatant was removed. The remaining crude protein suspension was adjusted to a pH of 7.0 with NaOH and then dialyzed against DI water at 4.0 °C for 36 h. Finally, the obtained API sample was freeze-dried for further analysis. The resulting API content was approximately 912 g/1000 g of powder with a nitrogen conversion factor of 6.25, as determined by the Kjeldahl method [29].

### 2.3. Characterization Analysis of APIs

#### 2.3.1. SEM Images of APIs

The microstructure of the API particles was observed using the method described by Liu and Tang [30], with minor modifications. SEM images of the APIs were obtained using an SEM (Thermo Fisher Scientific, Waltham, MA, USA) at an accelerating voltage of 30.0 kV and a magnification of 3000×. The APIs were affixed to a metal stand with a conductive adhesive and coated with gold before testing.

#### 2.3.2. Molecular Mass

The molecular weight of the APIs was measured according to the method in Fullington [31], with minor modifications. Sodium dodecyl sulfate-polyacrylamide gel electrophoresis (SDS-PAGE) was conducted with molecular weight markers ranging from 10 to 180 kDa. The protein samples were observed to migrate at a constant voltage of 80 V at a slow rate into the concentrating gel. Upon reaching the 5% demarcation line of the concentrating gel and the 12.5% demarcation line of the separating gel, the voltage was increased to a constant value of 120 V until the bands extended to the bottom of the gel. Subsequently, the gel was stained with a staining solution (composed of Kaumas Brilliant Blue R-250 (0.2 g), 95% ethanol (84 mL), glacial acetic acid (20 mL), and water (200 mL)) for 2–3 h. Subsequently, the gel was destained with a destaining solution composed of 450 mL of 95% ethanol, 50 mL of glacial acetic acid, and 500 mL of water. Subsequently, the APIs were observed and photographed using a gel imager.

#### 2.3.3. Fourier-Transform Infrared Spectroscopy (FT-IR)

Recording the FT-IR spectra of ultrasound was performed on the APIs using a spectrophotometer (FT-IR 8300, Shimadzu, Japan). The samples (10 mg) were mixed with KBr (200 mg) after drying to be ground and then pressed into thin slices. Absorption spectra were collected with a wave number range of 400 cm^−1^ to 4000 cm^−1^ at 25 °C; the resolution was 4 cm^−1^, and the scanning frequency was 32 times. Graphical analysis was performed using Ominc 9.2 and Peakfit v 4.12 software.

#### 2.3.4. Contact Angles

The contact angle of the APIs was analyzed with a contact angle measuring instrument (JGW-360A, Chengde Cheng hui test Co., Ltd., Chengde, China).

#### 2.3.5. Emulsifying Activity Index (EAI), Emulsifying Stability Index (ESI), Foaminess Index (FC), and Foam Stability Index (FS) of APIs

The APIs were dispersed in DI water at a concentration of 10 mg/mL. The protein sample solution was poured into soybean oil at a ratio of 7:3 (water to oil) by volume and homogenized with a homogenizer (XHF-DY, Ningbo Xinzhi Biotechnology Co., LTD, Ningbo, China). After homogenizing, 50.0 μL of the bottom emulsion was sucked and dispersed into 15 mL of 0.1% SDS solution immediately. At 0 and 10 min, the absorbance values at 500 nm were observed using a spectrophotometer (UV5, Mettler Toledo, Greifensee, Switzerland). The EAI and ESI were calculated using Equations (1) and (2), as follows:

(1) $$EAI=\frac{A_{0}\times N}{\psi \times L\times C\times 1000}$$

(2) $$ESI=\frac{A_{0}\times 10}{A_{0}-A_{10}}$$

where N is the dilution factor (100), T is a constant (2.303), L is the optical path (1.0 cm), Ψ is the volume fraction of oil phase (0.3), C is the concentration of protein before emulsion formation (10 mg/mL), and A_0_ and A_10_ are the absorbance value of the emulsion at 0 and 10 min, respectively.

According to the method by Tian [32], with slight modifications, 100 mL of 10 mg/mL sample was prepared, the pH was adjusted, the mixture was stirred at 8000 r/min for 2 min using a high-speed disperser and poured rapidly into a 500 mL measuring cylinder, and the volume of foam at the time of stirring cessation was calculated. Then, the total volume of liquid and foam at 10 min, 30 min, and 60 min was recorded.

(3) $$FC=\frac{V_{1}-V_{0}}{V_{0}}\times 100\%$$

(4) $$FS=\frac{V_{2}-V_{0}}{V_{0}}\times 100\%$$

Here, V_0_ is the sample volume in mL; V_1_ is the total volume of liquid and foam when stirring stops in mL; and V_2_ is the total volume of liquid and foam at 10 min, 30 min, and 60 min in mL.

### 2.4. Preparation and Characterization of Emulsions Stabilized by APIs

#### 2.4.1. Emulsion Preparation

First, the APIs were dispersed in DI water with 0.02% (*w*/*v*) sodium azide. The API suspensions were adjusted to different pH levels with HCl/NaOH. Then, the API suspensions were stirred and hydrated at 100 rpm for 2 h using a magnetic stirrer (IKA C-MAG HS 10, Staufen, Germany).

The soybean oil and the hydrated API suspensions with different pH levels were mixed at a ratio of 3:7. After that, the mixtures were sonicated using a high-intensity ultrasound processor (VCX-750, Sonics & Materials, Newton, CT, USA) at 40% pressure amplitude for 3 min in a pulse mode (3 s on and 3 s off) with an ice water bath. The resulting emulsion was stored at 4 °C before further analysis.

#### 2.4.2. Microstructure Observation

The microstructure images of the Pickering emulsion stabilized by APIs were observed using an optical microscope (ECLIPSE Ci-L, Nikon Inc., Tokyo, Japan) equipped with a digital camera (E3ISPM12000KPA, Hangzhou Toup Tek Photonics Co., Ltd. Hangzhou, China). The Pickering emulsion (10 μL) was deposited on the microscope slide. All the measurements were performed at 25 °C with a magnification of 400×.

The microstructure of the emulsions was also measured using a confocal laser scanning microscope (CLSM; FV-1000, Olympus Optical Co., Ltd., Tokyo, Japan). Briefly, the prepared emulsions (1.0 mL) were stained with 20 μL of Nile Red (0.02%, *w*/*v*) and 20 μL of Nile Blue (0.2%, *w*/*v*) and placed in darkness for 20 min. The dyed emulsions were placed on microscope slides and covered with coverslips. The CLSM images were obtained with an argon/krypton laser exciting at 488 nm for Nile Red and an He–Ne laser with excitation at 594 nm for Nile Blue.

Briefly, ethane gas (99.9% pure from BOC) was liquefied using liquid nitrogen [33]. Holey carbon grids were prepared as originally described [34]. The grids were glow discharged in a Denton Vacuum Evaporator (DV-501, Denton Vacuum Inc., Denton, TX, USA). A total of 2.0 µL of emulsion was loaded on hydrophilic grids, and excess liquid was removed by blotting before plunging the grids into liquid ethane cooled with liquid nitrogen. Loaded grids were immediately placed into liquid nitrogen to freeze. Frozen samples were immediately transferred into a cryo-scanning electron microscope (Cryo-SEM, Helios Nanolab G3 CX, FEI, Waltham, MA, USA). Images were recorded at a low temperature (about −175 °C) using 80 kV at a magnification of 20,000×.

#### 2.4.3. Droplet Size and Zeta Potential Measurements

A Malvern Instruments Zetasizer (Nano ZS Malvern Instrument Ltd., Worcs, UK) was used to measure the size and zeta potential of the emulsion droplets. First, 3.0 mL of the emulsion was dispersed into DI water (300.0 mL) to measure its average size and volume distribution. Next, the emulsions were diluted 500 times to observe their zeta potential. All samples were measured three times.

#### 2.4.4. Interface Adsorption Rate of Proteins

The protein adsorption rate calculation was described by Cai, with modifications [35]. A total of 8 mL of fresh PEs was centrifuged with 10,000× *g* for 10 min at 4 °C. The lower aqueous phase was collected using a syringe, and the concentrations of proteins in the aqueous phase were analyzed using Lowery’s method reagent. Finally, the percentage (%) of adsorbed protein was calculated.

#### 2.4.5. Rheological Measurements

A rotational rheometer (Mars III, Haake, Karlsruhe, Germany) equipped with a 35 mm-diameter parallel plate was used to characterize the rheological properties of the emulsions. The gap was set at 1 mm. The apparent viscosity was also recorded as the shear rate increased from 0.01 to 100 s^−1^ at 25 °C. The linear viscoelastic region (LVR) was determined in a strain range from 0.005% to 100%. The elastic modulus (G′) and loss modulus (G″) were tested within LVR as the oscillatory frequency sweep from 0.1 to 20 Hz at 25 °C.

#### 2.4.6. Creaming Index (CI) Analysis of APIs

The CI can also indicate the stability of emulsions [36]. The fresh emulsions (10 mL) were poured into the glass bottles (25 mL) at 4 °C. The CI was determined at day 0 and day 20 using Equation (5).

(5) $$CI=\frac{Hs}{Ht}\times 100\%$$

Here, Hs is the height of the serum, and Ht is the total height of the emulsion.

### 2.5. Encapsulated Astaxanthin

#### 2.5.1. Preparation of Astaxanthin-Loaded Emulsions

An amount of astaxanthin was first completely dissolved in soybean oil, and then it was mixed with a suspension of API at a concentration of 10 mg/mL in a 3:7 ratio. Finally, the mixture was sonicated with a high-intensity ultrasonic processor (VCX-750, Sonics & Materials, Newton, CT, USA) in pulse mode at a 40% pressure amplitude for 3 min (3 s on, 3 s off), and the entire sonication process was carried out in an ice water bath. The resulting emulsion was stored at 4 °C and protected from light until further analysis.

#### 2.5.2. Color Determination

The color of the astaxanthin-loaded emulsions was measured at 25 °C with a handheld Minolta Chroma-meter^®^ CR-400 (Osaka, Japan) using an 8 mm aperture illuminant D65 (6500 K color temperature) at a standard observation of 2°. The colorimeter was calibrated using a standard white background (CIEL* = 97.49, a* = −0.21, b* = 0.67; Minolta calibration plate). CIE-LAB parameters (lightness, L*, redness, a*, and yellowness, b*) were measured at the three-point surface of the samples. Each sample was measured 3 times.

#### 2.5.3. Determination of Astaxanthin Encapsulation Efficiency

The embedding efficiency in the emulsion was determined spectrophotometrically using the method by Saechio [24], with some modifications. Unembedded astaxanthin was extracted with hexane. A total of 0.2 g of emulsion was weighed, 20 mL of hexane was added and mixed well, the mixture was centrifuged to collect the clear liquid, and then it was diluted a certain number of times and stored away from light. A mixture of 2 mL of hexane and 1 mL of ethanol was used to extract all the astaxanthin in 0.20 g of emulsion, and the absorbance value of astaxanthin was measured at 475 nm in the extract. The corresponding astaxanthin content was calculated according to the standard curve of astaxanthin. The astaxanthin encapsulation efficiency (AP%) was calculated as follows:

(6) $$AP\%=\frac{T_{1}-T_{2}}{T_{2}}\times 100\%$$

where T_1_ is the total concentration of astaxanthin in the emulsion in mg/mL, and T_2_ is the concentration of extracted unembedded astaxanthin in mg/mL.

### 2.6. Statistical Analysis

Data were analyzed using a one−way analysis of variance (ANOVA). Means were compared with the Duncan multiple-range test at *p* < 0.05 using SPSS 26.0 software. Each treatment was replicated three times. Results were presented as mean ± SD.

## 3. Results and Discussion

### 3.1. Characterization of APIs

#### 3.1.1. Morphology of API

**Figure 1:**
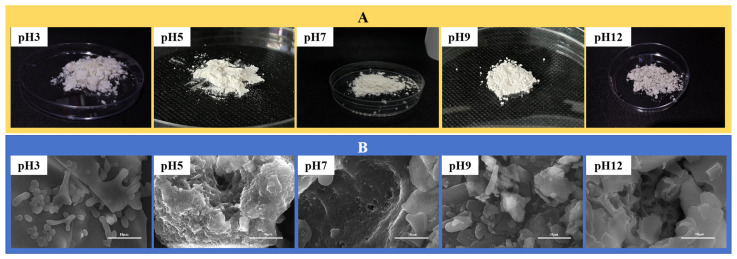
(**A**) Appearance of APIs at pH levels of 3, 5, 7, 9, and 12, passing through a 200 mesh sieve; (**B**) SEM micrographs of the APIs at pH levels of 3, 5, 7, 9, and 12. The scale bars are 10.0 μm.

In the SEM image (Figure 1), it can be seen the API aggregates are the largest at a pH of 5, and the API has a rough surface and irregular fluffy appearance. At this pH, the positive and negative charges on the protein surface are close to zero (our previous experiments revealed that a pH of approximately 4.7 was the isoelectric point of the API), the electrostatic repulsion is the smallest, the API solubility is the lowest, and the protein particle aggregates are easily precipitated. At a pH of 3, the API appears as a smooth and uniform ellipsoid, which may be attributed to the breaking of hydrogen bonds within the protein particles under strongly acidic conditions, leading to the breakdown of large molecular structures into small molecular structures. At a pH of 12, the API surface becomes smooth and porous. Rice bran-modified wheat gluten nanoparticles also show similar results, with wheat gluten nanoparticles aggregating into irregular flocs at the micrometer level [37]. Similar phenomena were observed in a study of quinoa protein isolate [38]. Therefore, pH is an important factor affecting the structure of protein particles.

#### 3.1.2. Structure of the API

**Figure 2:**
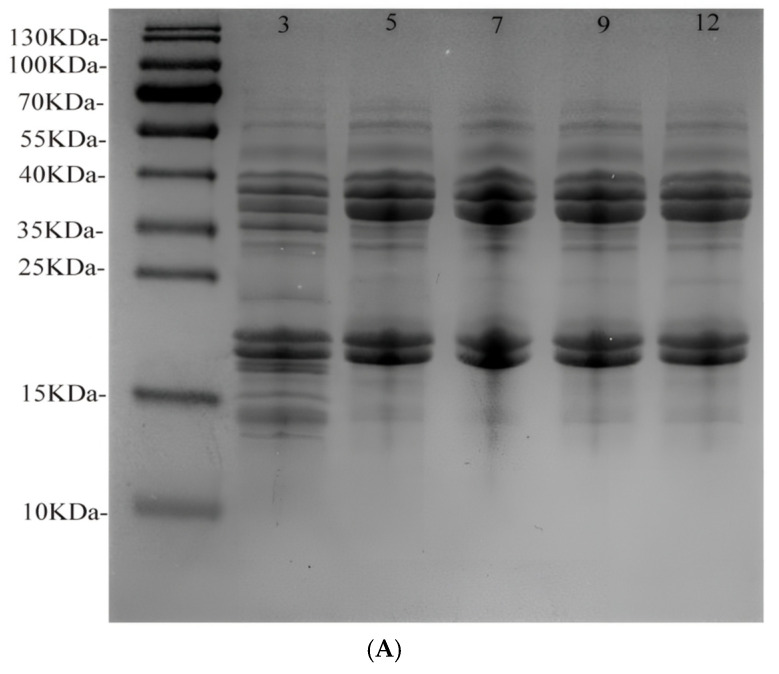

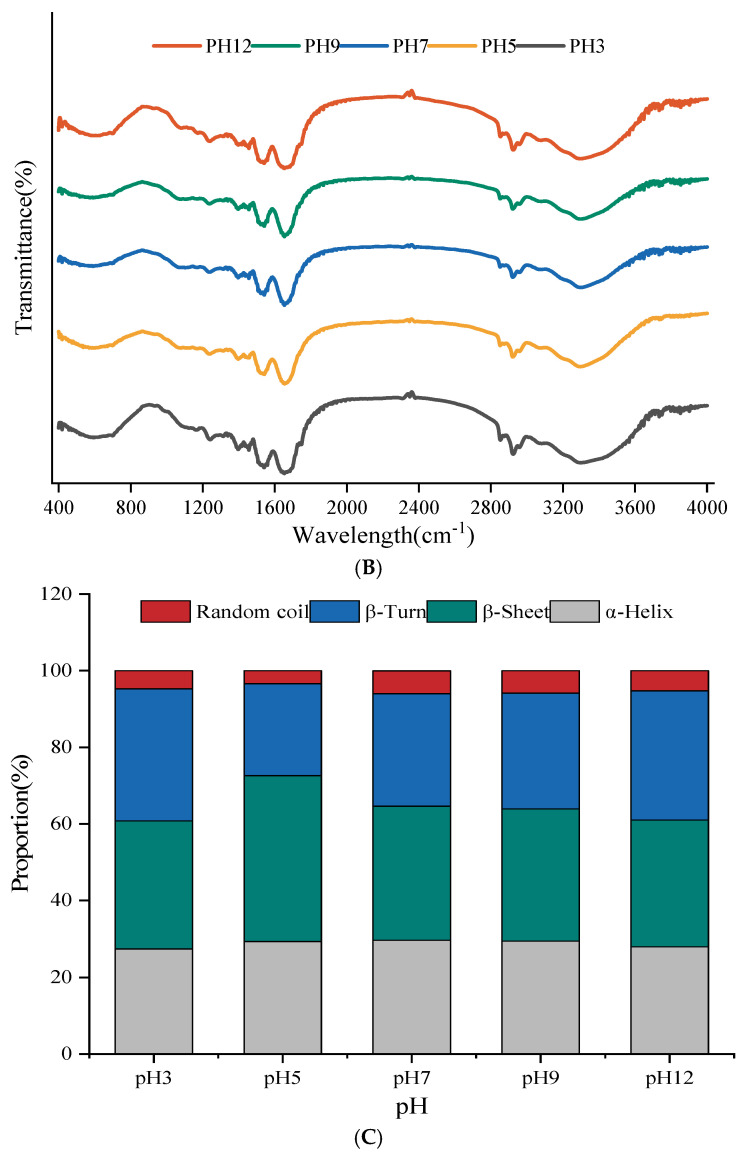
SDS−PAGE (**A**), FTIR (**B**), and secondary structure content (**C**) of APIs at pH levels of 3, 5, 7, 9, and 12.

Under reducing conditions, the electrophoretic profiles of the API were significantly different at different pH values (Figure 2A). At a pH of 3, the API showed multiple bands between 15 kDa and 40 kDa, where disulfide bonds within the protein were broken and disassembled into polypeptides, suggesting that a pH of 3 may cause API breakage. However, the intensity of the electrophoretic bands for the API did not vary significantly at other pH values. Sathe et al. explained that the addition of mercaptoethanol under reducing conditions breaks the disulfide bonds between peptide complexes and produces small-molecule peptides [39]. Therefore, the molecular distribution of protein samples did not change at other pH values, except at a pH of 3.

Figure 2B shows the characteristic absorption peaks of the proteins in the NIR region, which can be used to analyze their secondary structure. All samples exhibited a broad stretching vibration peak at 3700–3200 cm^−1^, and the variation in this characteristic absorption peak was related to the variation in the number of hydroxyl groups [40]. When the API was in a strongly acidic (pH 3) or basic (pH 12) environment, the absorption peaks broadened at 3700–3200 cm^−1^, indicating that strong acids or bases increase the number of hydroxyl groups in the proteins, leading to vibrational absorption, which breaks the hydrogen bonds between the proteins and may denature them. Changes in the characteristic absorption peaks at absorption wavelengths of 1600–1700 cm^−1^, 1530–1550 cm^−1^, and 1260–1330 cm^−1^ represent amides I, II, and III, respectively [41]. The amide III band depends on the force field, side chains, and hydrogen bond properties. Notably, Figure 2B and Table 1 show that the peak intensity of amide III absorption in the API was significantly greater at pH levels of 3 (0.042) and 12 (0.046) than it was at a pH of 5 (0.014). Owing to the differences in the half-peak widths, intensities, and peak positions of the spectral bands at different pH values, it is hypothesized that hydrophobic interactions, hydrogen bonding, and electrostatic interactions between protein molecules at different pH values may affect the secondary structure of the proteins [42]. According to the peak-fitting analysis, the relative level of β-folding in the secondary structure of the protein was highest at a pH of 5 (43.23%; close to the isoelectric point of amygdalin) (Figure 2C), and the relative level of β-folding at pH levels of 3 and 12 was significantly lower than that at a pH of 5. The relative level of β-turning was highest at a pH of 3 (34.48%), followed by that at a pH of 12 (33.76%), which was significantly higher than that at a pH of 5 (*p* < 0.05), indicating that the API structure changed from ordered to disordered and became more relaxed when the pH was far from the API isoelectric point [43]. In addition, the β-folded sheet content correlated with the hydrophobic interactions of the protein, with a decrease in hydrophobic interactions indicating the exposure of hydrophobic sites within the molecule and increased surface hydrophobicity, a speculation that is consistent with that in Section 3.1.4. The relative content of α-helices was 27.40% at a pH of 3, which was significantly lower than that at a pH of 5 (*p* < 0.05) because of the increase in electrostatic effects on the protein molecule charge, which affected the stabilization of hydrogen bonding.

#### 3.1.3. Emulsification Activity Index (EAI), Emulsification Stability Index (ESI), Foaming Capacity (FC), and Foaming Stability (FS)

The EAI characterizes the solubility of protein particles in the aqueous phase and their ability to strongly adsorb onto the oil–water interface to form an emulsion layer. The ESI characterizes the stabilizing ability of an emulsion to form small droplets [44]. At a pH of 5, the API had the lowest EAI and ESI values; at a pH of 3, the API had the highest EAI and ESI values (Table 2). The API had the highest ESI value at a pH of 12 (98.50 min). Therefore, at pH levels of 3 or 12, the API favors the formation of an interfacial layer at the oil–water interface, which prevents the aggregation of emulsion droplets and enhances emulsion stability.

Protein foam is composed of numerous air droplets wrapped in a protein film, which is generally determined by the surface tension magnitude; a lower surface tension is more favorable for foam formation. The FC and FS indices of the API were significantly different at different pH values (Table 2). A pH of 5 (near the isoelectric point) showed the lowest FC and FS indices of the API; the FS index was close to zero, and the foam disappeared after 1 h. The FC indices of the API at pH levels of 3 and 12 were as high as 83.80% and 105.70%, respectively, which were significantly higher than those at a pH of 5 *(p* < 0.05), and the FC indices, at 30%, were significantly higher than that at a pH of 5 (*p* < 0.05). After 1 h of standing, the FS index of the API at a pH of 3 decreased from 83.80% to 43.37%, with a decrease of 48.25%, and that of the API at a pH of 12 decreased from 105.70% to 43.80%, with a decrease of 58.44%, suggesting that the API at a pH of 3 had the best foam stability, with its emulsification facilitated by its low surface tension at the oil–water interface.

#### 3.1.4. Contact Angle

**Figure 3:**
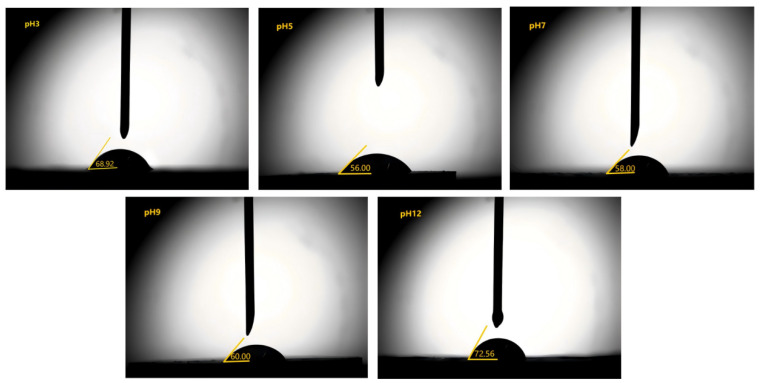
Contact angles (θ) of APIs at different pH values.

**Figure 4:**
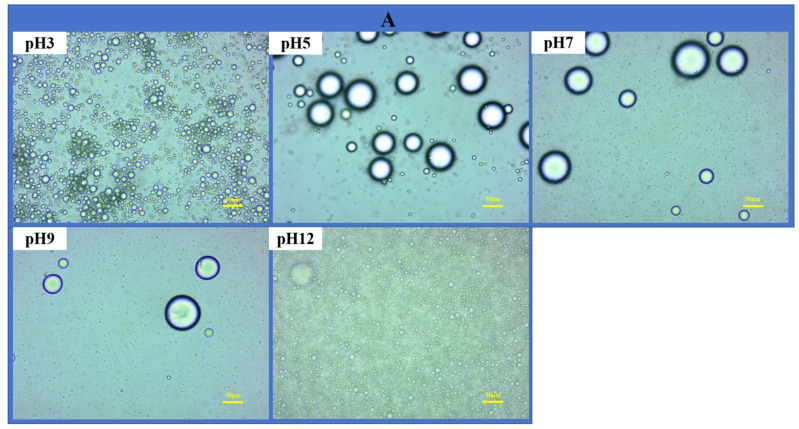

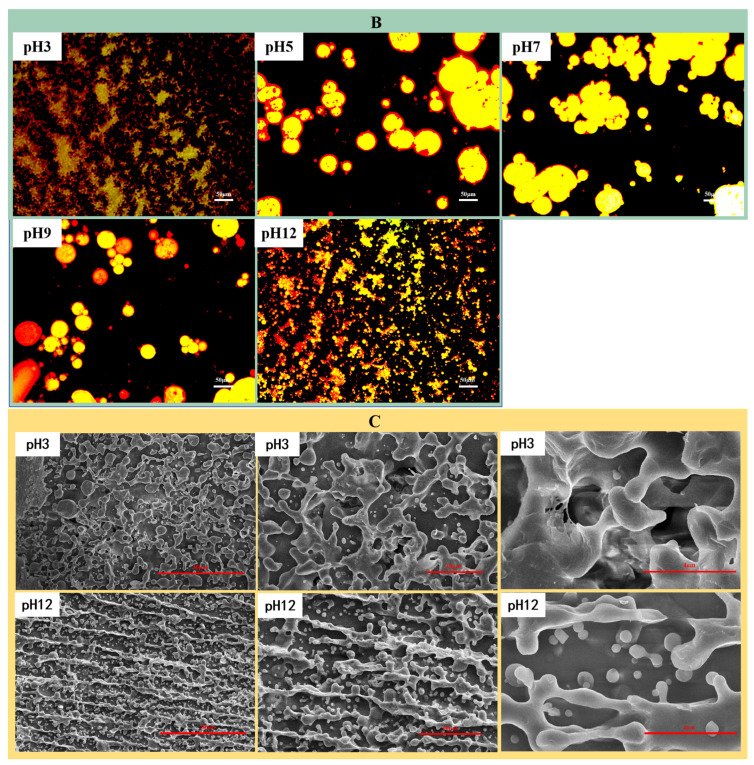
OM (**A**) and CLSM images (**B**) of stabilized emulsions with an API concentration of 1.0 wt%. (**C**) The Cryo−SEM image of stabilized emulsions with an API concentration of 1.0 wt% at pH levels of 3 and 12. The volume fraction of the oil was controlled to 30%.

The contact angle usually characterizes the wettability of the particles and is an important parameter for determining the type and stability of an emulsion [6]. The maximum desorption energy E of solid particles at the oil–water interface is at an angle of 90°, and particles with θ *<* 90° are predominantly hydrophilic when immersed in the aqueous phase and form oil-in-water emulsions. Water-in-oil emulsions were obtained using hydrophobic particles with θ *>* 90°. The contact angles of the APIs at different pH values are shown in Figure 3. The contact angles of APIs at pH levels of 3, 5, 7, 9, and 12 were 68.92°, 56.00°, 58.00°, 60.00°, and 72.56°, respectively. The contact angles of APIs at different pH values were less than 90°; thus, the API particles showed typical hydrophilicity and formed oil-in-water emulsions, which is a result that is consistent with the CLSM (Figure 4B). In contrast to the amphiphilicity of protein molecules, the wettability of particles is the main factor determining the stabilization mechanism of PEs. Previous studies have shown that as the pH moves away from the isoelectric point of proteins, the hydrophobic interactions between protein particles in emulsions weaken, and electrostatic mutual repulsion increases; hence, they can better adhere to the oil–water interface to form an interfacial film that prevents droplet aggregation and improves emulsion stability. Buckley et al. have shown that the optimal θ for the formation of protein particles into monolayers at the air–water interface is 71° [45], from which it can be inferred that API particles form a tightly packed monolayer interfacial barrier at the oil–water interface at pH levels of 3 and 12.

### 3.2. Characterization of API-PE

#### 3.2.1. Microstructure

The microstructure of an emulsion, such as its interfacial structure and particle position, plays a dominant role in its physical stability [46]. Under an optical microscope, the size of the emulsion droplets increased and then decreased with increasing pH (Figure 4A). The emulsion droplets were the largest and were not uniformly distributed at a pH of 5. At pH levels of 3 and 12, the size of the emulsion droplets decreased, and they were uniformly distributed.

Figure 4B shows the CLSM images of the emulsions obtained at different pH values. Red fluorescence indicates the API in the continuous phase, and yellow fluorescence indicates oil droplets in the discontinuous phase. These results confirm that the oil droplets were encapsulated by the API particles to form oil-in-water PEs. For example, Zhang et al. investigated the stabilization of PEs with pea protein isolate–chitosan nanoparticles, and CLSM images confirmed that the solid particles encapsulated the oil droplets by forming an interfacial layer film at the oil–water interface [47]. In addition, droplet size variation with pH observed using CLSM was consistent with that observed using optical microscopy. These results indicate that API PEs at pH levels of 3 and 12 are stable, preventing aggregation between droplets.

Based on the aforementioned studies, we analyzed emulsions at pH levels of 3 and 12 using Cro-SEM. At a pH of 3, the API adhered to the surface of the oil droplets and formed an irregular three-dimensional network structure (Figure 4C). This can be attributed to the breaking of non-elliptic bonds that maintain the natural conformation of the protein and the exposure of hydrophobic residues on the side chain surfaces, causing the protein peptide chains to unfold, thereby generating more energy to cross-link the excess API in the aqueous phase and stabilize the network structure of the emulsion. At a pH of 12, the microscopic structure of the emulsion formed a regular three-dimensional network, which can be explained by the negative surface charge of proteins in strongly alkaline environments. This leads to the departure of the corresponding positively charged ligands interacting with the proteins from their surfaces, as well as the formation of aligned layers of anchored orientation at the oil–water interface through the action of permanent dipoles and hydrogen bonding.

#### 3.2.2. Droplet Size and Zeta Potential

**Figure 5:**
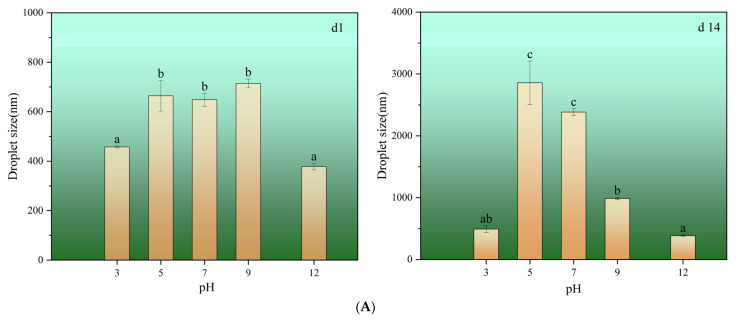

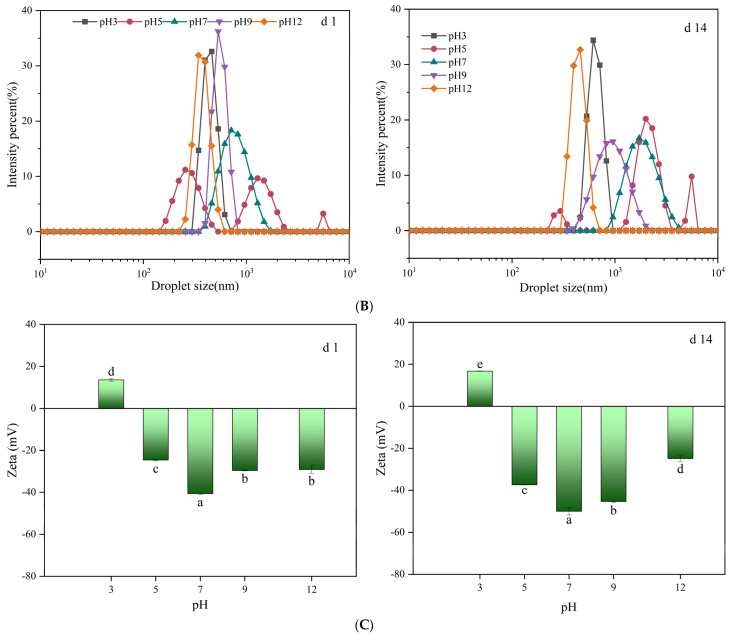
Droplet size (**A**), percentage size (**B**), and zeta potential (**C**) of API−stabilized PEs at different pH values (3–12) on day 1 and day 14. The results were expressed as mean ± standard deviation (n = 3). a–e: Different letters above standard deviation bar indicate significant differences among the means (*p* < 0.05).

pH is a key factor influencing flocculation in emulsions [48]. Emulsion instability is characterized by flocculation, agglomeration, delamination, and Ostwald ripening. Uneven droplet dispersion is the main reason for Ostwald ripening, which is closely related to the particle size distribution [3]. According to Stokes’ law [49], decreasing the size of the emulsion droplets and/or increasing the emulsion viscosity can inhibit gravitational segregation, thereby keeping the emulsion stable. Figure 5A shows that different pH values have different effects on the droplet size. The average droplet size of PEs were 457.5 nm, 664.3 nm, 648.2 nm, 614.2 nm, and 378.1 nm at pH levels of 3, 5, 7, 9, and 12, respectively, on day 1. The average droplet size of the emulsion was the largest at a pH of 5, resulting in flocculation and decreased interfacial-specific surface area, which was not conducive for API adsorption at the oil–water interface, with the macroscopic morphology of the emulsion showing the same effect. The macroscopic morphology showed that the phase separation of the emulsions occurred on day 1. This is because the flocculation of emulsions in the vicinity of pI is mainly caused by weakened electrostatic interactions between droplets. Sriproblom et al. investigated whey protein-stabilized oil-in-water emulsions and obtained similar results [50]. The droplet sizes of the emulsions at pH levels of 3 and 12 did not change significantly after 14 days, indicating relatively stable adsorption of the API in the emulsions at the oil–water interface. However, unlike previous studies on pH-adjusted pea isolate protein-stabilized emulsions, the droplet size of the emulsion droplets increased, and the stability of the emulsions decreased at lower pH levels [51].

Droplet size distribution can be used as a judgment indicator of the degree of uniformity of PEs; the more concentrated the droplet size distribution range, the more dispersed and uniform the droplet distribution of PEs. The average droplet size distribution of the emulsion at a pH of 5 on day 1 was multi-peaked. On day 14, the peak width increased. In contrast, the peak value decreased, with the droplet size distribution ranging from 164.2 nm to 5560 nm (Figure 5B), which was presumed to be determined by the nature of the protein surface charge. Close to the isoelectric point of proteins, the electrostatic interactions weakened, resulting in droplet aggregation; thus, the emulsions at a pH of 5 became extremely unstable, and macroscopically, the droplet distribution of PEs was more homogeneous [47]. Therefore, the emulsion was extremely unstable at a pH of 5. Macroscopically, a large amount of oil precipitated from the upper layer of the emulsion after 14 days. The droplet size distributions of the emulsions at pH levels of 5, 7, and 9 all shifted to the right on day 14. However, the droplet size distribution of the emulsions at pH levels of 3 and 12 did not change significantly, and the API adhered better to the oil–water interface, forming a stable ground film.

The zeta potentials of the emulsions decreased with increasing pH levels. The zeta potentials of the emulsions at pH levels of 3, 5, 7, 9, and 12 were 39.07 mV, −4.47 mV, −16.87 mV, −28.97 mV, and −49.90 mV, respectively, on day 1 (Figure 5C). The absolute values of the zeta potential of the emulsions were all less than 30 mV at pH levels of 5, 7, and 9, and the electrostatic repulsions between the droplets were small and insufficient to resist the van der Waals forces between them. The API formed an unstable ground boundary mask, and the droplets were prone to polymerization with each other. van der Waals forces between the droplets were too small to resist, with the API forming an unstable ground boundary mask and the droplets polymerizing with each other. The emulsion at a pH of 12 had the largest absolute zeta potential value and was left for 14 days without phase separation. This may be attributed to the increased OH concentration in the solution, which readily binds to hydrogen atoms to form hydrogen bonds that are firmly adsorbed on the surface of emulsion droplets to form an oriented arrangement of hydration layers [46]. Delahaije et al. investigated the effect of charge on flocculation-induced protein-stabilized emulsions at different pH values and obtained consistent results [52]. The zeta potential of all emulsions decreased after 14 days of placement. Notably, the absolute zeta potential value of the emulsions at pH levels of 3 and 12 was still greater than 30 mV, the number of charges on the surface of the APIs increased, the stability of the charges carried by the APIs increased, and the mutual electrostatic repulsion increased, preventing droplets from agglomerating or polymerizing [53].

#### 3.2.3. Interfacial Protein Adsorption Rate

**Figure 6:**
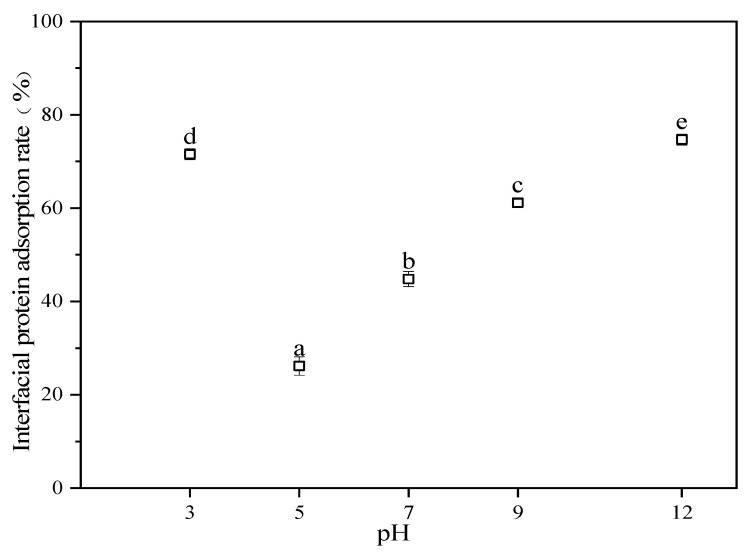
Interfacial protein adsorption rate of API-PEs at different pH values. The concentration of APIs was 1% *w*/*v*, and the fraction of oil was 0.3. The results were expressed as mean ± standard deviation (n = 3). a–e: Different letters above standard deviation bar indicate significant differences among the means (*p* < 0.05).

The physical stability of emulsions depends on the interfacial adsorption properties of proteins, owing to their ability to form a physical barrier at the oil–water interface, preventing droplets from approaching each other. A quantitative analysis of proteins adsorbed at the oil–water interface was used to study their actual adsorption in the dispersed phase in emulsions (Figure 6). The lowest interfacial protein adsorption rate of 26.18% was observed in emulsions at a pH of 5 because close to the isoelectric point of the APIs, the net charge on the surface of the protein particles and the electrostatic repulsive force between the APIs decreased. This was insufficient to overcome the hydrogen bond and hydrophobic interaction of the attraction; thus, the droplets flocculated, and the average particle size increased. On the right side of the isoelectric point, interfacial protein adsorption increased with increasing pH levels. On the left side of the isoelectric point, interfacial protein adsorption decreased with increasing pH, with the change rule similar to the solubility of the “U” law. The larger the interfacial protein adsorption, the stronger the interfacial protein adsorption energy and emulsification performance. The interfacial protein adsorption rates in the emulsion at pH levels of 3 and 12 were 71.56% and 74.69%, respectively, which were significantly higher than that in the vicinity of the isoelectric point of the API (*p* < 0.05). The interfacial protein adsorption amount was larger, and the interfacial membrane film was formed with increasing thickness, preventing the droplets from approaching each other. The increase in interfacial protein adsorption increased the thickness of the interfacial membrane layer and prevented the droplets from approaching each other. The interfacial adsorption of proteins decreases interfacial energy, the rearrangement of protein conformation, and assembly into viscoelastic two-dimensional nanomembranes [54]. Zhang et al. stabilized oil-in-water PEs using pea protein gel particles and found that away from the isoelectric point of pea protein, the interfacial protein adsorption of the emulsion increased, the emulsion stability improved protein adsorption, and emulsion stability improved. These findings are consistent with those in this chapter [43].

#### 3.2.4. Rheology

**Figure 7:**
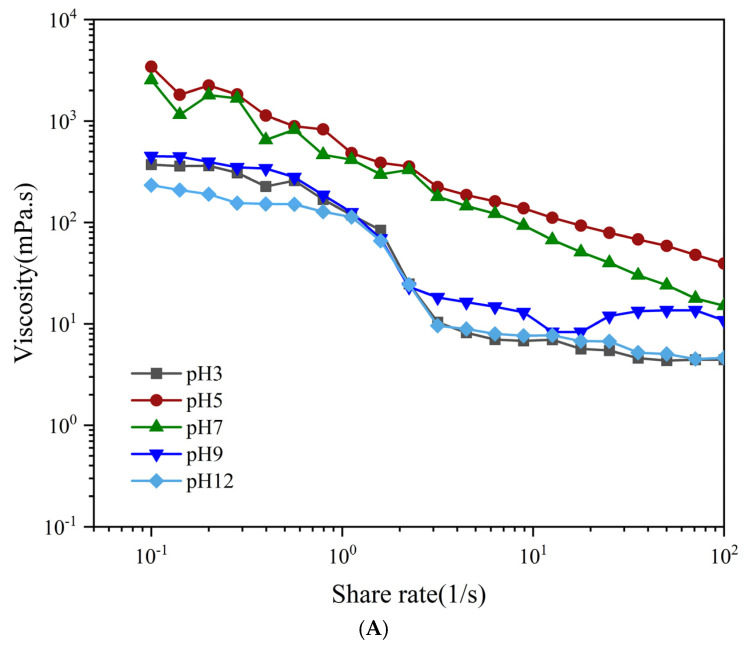

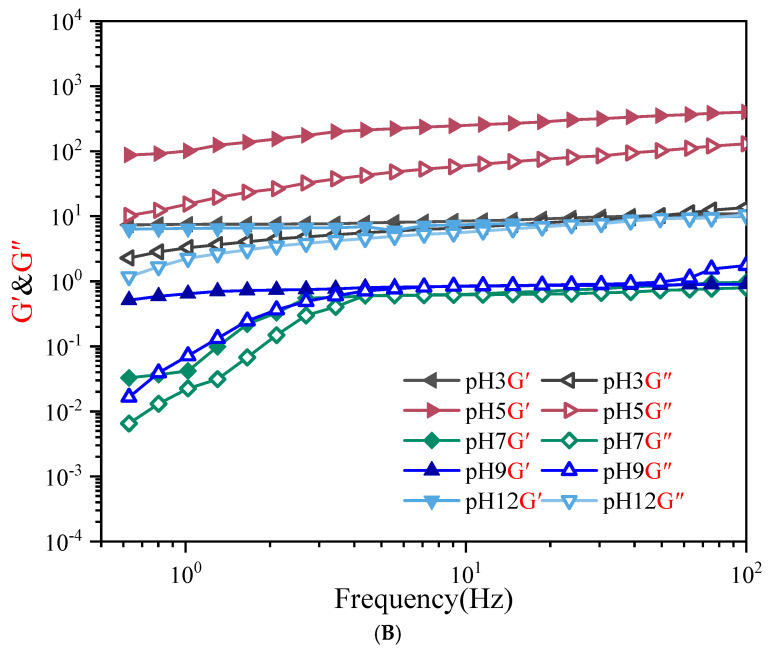
Rheological properties (**A**) viscosity, (**B**) frequency of API−PE at different pH values. The concentration of APIs was 1% *w*/*v*, and the fraction of oil was 0.3.

The physical stability of emulsions is related to their rheological properties as a function of viscosity. When the shear rate (in the range of 0.01 s^−1^–100 s^−1^) is the same, the viscosity of all samples decreases with the increasing shear rate, exhibiting typical non-Newtonian pseudoplastic behavior (shear thinning) (Figure 7A). The viscosity of PEs decreases with the increasing shear rate because the increase in shear rate overcomes the combined effect of Brownian motion between droplets and the weak bonding between proteins or proteins and fats. The droplets are ordered along the streamlined direction, and the inter-droplet friction decreases. Throughout the test, the apparent viscosity of the emulsion at pH 5 was always the highest, the friction at the oil–water interface increased, and the movement of the API at the oil–water interface reduced, resulting in a higher resistance to flow within it, which increased the apparent viscosity of the emulsion. The apparent viscosity of the emulsions at pH levels of 3 and 12 decreased, the friction of the API at the oil–water interface decreased, and the movement of the API at the oil–water interface accelerated. Zhang et al. used peas to prepare emulsions and found that flocculation of the emulsion droplets occurred close to the isoelectric point of the pea proteins, and the apparent viscosity of the emulsions was enhanced. Similar findings have been reported for pineapple honey seed protein-stabilized PEs [47].

The energy storage modulus (G′) and loss modulus (G″) can be used to assess the flocculation of emulsion systems. Figure 7B shows that G′ and G″ increase with increasing frequency over the entire frequency sweep (0.1 Hz–100 Hz). For PEs at a pH of 5, G′ is greater than G″, forming an elastic weak gel structure, with electrostatic repulsion between proteins being less than hydrophobic interactions, and mutual attraction such as hydrogen bonding resulted in API aggregation and uneven distribution at the oil–water interface, which is consistent with the observation in CLSM. This contrasts with Hu’s findings on the formation of stable PEs using colloidal particles of alcohol-soluble proteins, in which the emulsions were more stable at pH values close to the isoelectric point of alcohol-soluble proteins [55]. pH values far from the isoelectric point of the APIs resulted in gel emulsions that formed during the increasing initial frequency, where G′ was greater than G″, and as the vibrational frequency increased, the modulus value to the intersection point G″ became larger than G′, and the emulsion system became a liquid fluid dominated by viscous behavior. This indicates that increasing the oscillation frequency exacerbates the irregular movement of droplets, and the non-covalent interactions existing between molecules and droplets become weak, destroying the weak gel network structure of the emulsion [56].

#### 3.2.5. Creaming Index (CI)

**Figure 8:**
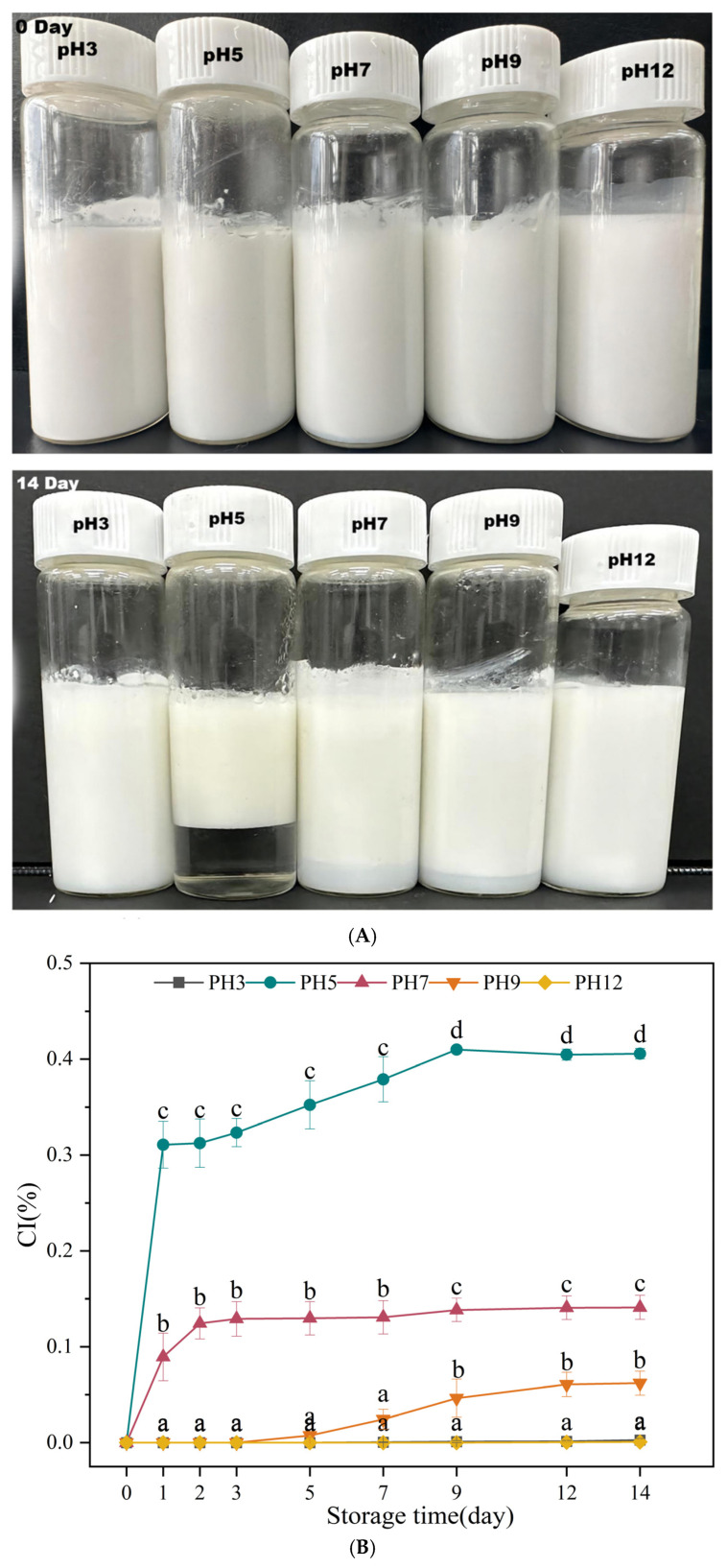
(**A**) Typical visual images of the various emulsions stored for 14 days. (**B**) Creaming index of API-PEs during 14 days of storage at different pH values. The API concentration was 1.0% *w*/*v*, and the oil fraction was 0.30. The results were expressed as mean ± standard deviation (n = 3). a–d: Different letters above standard deviation bar indicate significant differences among the means (*p <* 0.05).

The CI of the API-PEs formed at different pH values with a fixed oil phase fraction (0.3, *v*/*v*) in static storage for up to 14 days was also evaluated. As expected, the phase separation behavior of these emulsions highly correlated with the aqueous-phase pH. The CI values of all emulsions were shown to increase and then decrease with increasing pH (3–12), which increased with longer storage time (Figure 8A). Similar emulsification behavior has been observed for PEs stabilized with soy protein nanoparticles. On day 1 of storage, the CI of the emulsion at a pH of 5 was 31.1% (maximum), with severe emulsion delamination and weakened electrostatic mutual repulsion between the API molecules. The van der Waals and hydrophobic interaction mutual attraction accelerated the aggregation of emulsion droplets, and the average size of the emulsion droplets increased, with the emulsion undergoing phase separation owing to gravitational force according to Stokes’ law. As the storage time increased, the molecular motion in the emulsion intensified, leading to the rearrangement or shedding of proteins adsorbed at the oil–water interface. Simultaneously, the gravity and Brownian transport of the particles resulted in decreased emulsion stability. The emulsion emulsification index changed the most at a pH of 5 on day 14 (40.57%). The electrostatic interactions between the APIs distributed at the oil–water interface were not sufficient to overcome gravity, and the aqueous phase became transparent, indicating that the particles were adsorbed at the oil–water interface. Some of the particles migrated to the oil phase, and oil leakage and emulsification occurred. The electrostatic repulsive interactions between the APIs were strengthened at pH levels of 3 and 12, overcame gravity, and formed a stable interfacial film, which was then stabilized by gravity. At pH levels of 3 and 12, the electrostatic repulsion between the APIs increased, overcoming gravity and forming a stable interfacial film, with no phase separation occurring in the emulsion.

### 3.3. Embedding AST

Table 3 shows the effect of different pH values on the average droplet size, color parameters, and encapsulation rate of emulsions loaded with AST. The average droplet size of the emulsions varied significantly at an AST concentration of 0.05% (*w*/*v*), an oil phase volume fraction of 0.30, an API concentration of 2.0%, and aqueous-phase pH levels of 3, 5, 7, 9, and 12. A pH of 5 showed the maximum average droplet size of 733.8 nm. The droplet size of the emulsions was reduced by 33.54% at a pH of 3 compared to that at a pH of 5, and the interfacial-specific surface area increased. The API adsorption at the oil–water interface increased, and an interfacial film was formed to prevent the droplets from coming into contact with each other. AST was encapsulated in the oil phase, thus preventing AST oxidation in the oil phase. The encapsulation rate of AST in the emulsion increased by 65.16%. The emulsion particle sizes at pH levels of 7, 9, and 12 were 686.1 nm, 711.2 nm, and 471.2 nm, respectively, which was significantly lower than that of the emulsion droplets at a pH of 5 (*p* < 0.05). When the pH was far from the isoelectric point of the API, the API charge in the emulsion increased, and the electrostatic repulsion between droplets improved, which prevented droplets from approaching each other. The emulsion stability also improved, which led to an increase in the encapsulation rate of AST. At a pH of 12, the particle size of the emulsion decreased by 35.79%, and the encapsulation rate of AST increased by 55.64%. The preparation and characterization of Pickering latex stabilized with a whey-soluble protein/starch complex for the delivery of AST outlined by Song et al. showed a decrease in the particle size of the droplets and an increase in AST encapsulation [57].

**Figure 9:**
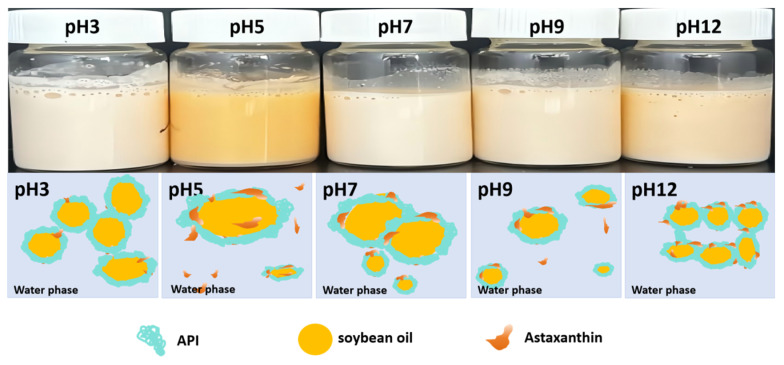
Appearance and schematic representation of AP-PEs loaded with astaxanthin at different pHs.

In Table 3, the a* value of the emulsion at a pH of 5 was highest at 12.28, and the emulsion had the darkest bright orange color because of AST oxidation in the partially encapsulated oil phase. When the pH was increased from 7 to 12, the a* value of the emulsion increased from 5.86 to 7.86, and the color of the emulsion gradually deepened (Figure 9). The structure of the AST molecule, especially its multiple double bonds, makes it easy to undergo free radical reactions in an oxidative environment, resulting in the oxidative decomposition of AST molecules. This destroys the original AST structure, forming a variety of different oxidation products, more commonly known as levorotatory or dextrorotatory structures. When the pH increased from 7 to 12, the a* value of the emulsion increased from 5.86 to 7.86, and the emulsion gradually deepened. At a pH of 3, the a* value of the emulsion was significantly lower than that at a pH of 5, and the emulsion was lightest in color because the protein particles at the oil–water interface aggregated to form an interfacial membrane, preventing AST in the oil phase from diffusing into the aqueous phase and reducing its binding to oxygen, thus protecting it from oxidation. The structure of AST distributed at the interface changes from trans to cis [58]; therefore, the emulsion color deepened to light orange at a pH of 12.

## 4. Conclusions

API-stabilized PE represents an advancement over traditional stabilization strategies that rely on surfactants and chemically synthesized emulsifiers; these traditional stabilization strategies have been associated with concerns regarding biocompatibility, toxicity, and environmental impact. Our findings demonstrate that API-stabilized PE can be an effective potential carrier for astaxanthin with pH responsiveness. This study investigated the correlation between the molecular structure and emulsification properties of API at different pH values and the API distribution at the oil–water interface. The results showed that at a pH of 5 (close to the API isoelectric point), the secondary API structure had the highest sheet content and lowest steering content, with the worst emulsification performance. Away from the API isoelectric point, the sheet content was low, the steering content was high, the emulsification performance was enhanced, and the hydrogen bonding that maintains the molecular structure changed, which loosened the API structure and increased the contact angle. The contact angle of the API at pH levels of 3 and 1 further proves that the API unfolds at the oil–water interface and forms a monolayer-arranged interfacial film. CLSM and Cro-SEM showed that the API was adsorbed at the oil–water interface and formed a dense packing layer with a three-dimensional mesh structure between the droplets, which was in a more regular three-dimensional mesh structure at a pH of 12, whereas the opposite was true at a pH of 3. On day 14 of storage, the droplet size of the PEs remained small and uniform at pH levels of 3 or 12 and showed good stability. The rheological properties of API-PE are closely correlated with changes in pH. API-PE is an effective delivery system for AST, as the encapsulation rate of AST significantly increases, which helps to tailor in vitro gastrointestinal tract excavation.

## Figures and Tables

**Table 1:** Frequency distribution and intensity of the characteristic Fourier-transform infrared spectrum (4000–400 cm^−1^) of the raw material treated with ultrasound at pH values of 3, 5, 7, 9, and 12.

pH		Amid I	Amid II	Amid III	Amid A
3	Frequency	1651	1539	1245	3273
	Intensity	0.327	0.299	0.042	0.072
5	Frequency	1653	1541	1243	3273
	Intensity	0.295	0.211	0.014	0.084
7	Frequency	1655	1541	1243	3274
	Intensity	0.135	0.183	0.016	0.059
9	Frequency	1652	1541	1241	3273
	Intensity	0.18	0.183	0.014	0.059
12	Frequency	1652	1539	1244	3269
	Intensity	0.431	0.399	0.046	0.17

**Table 2:** Effect of different pH levels on EAI, ESI, FC, and FS of APIs.

pH	EAI/(m^2^/g)	ESI/min	FC/%	FS/min		
				10 min	30 min	60 min
3	76.98 ± 0.22 ^d^	97.81 ± 0.03 ^c^	83.80 ± 1.35 ^d^	83.80 ± 1.35 ^d^	79.63 ± 1.29 ^d^	43.37 ± 0.74 ^d^
5	18.38 ± 1.34 ^a^	60.96 ± 4.49 ^a^	24.53 ± 2.48 ^a^	21.20 ± 2.63 ^a^	3.37 ± 0.15 ^a^	0.20 ± 0.35 ^a^
7	31.66 ± 4.00 ^b^	73.11 ± 1.78 ^b^	41.83 ± 1.84 ^b^	39.23 ± 1.15 ^b^	12.33 ± 1.01 ^b^	2.23 ± 081 ^b^
9	28.30 ± 0.09 ^b^	76.77 ± 3.15 ^b^	62.90 ± 1.08 ^c^	61.13 ± 1.76 ^c^	38.53 ± 1.45 ^c^	17.53 ± 2.32 ^c^
12	64.68 ± 0.09 ^c^	98.50 ± 0.07 ^c^	105.70 ± 2.60 ^e^	105.70 ± 2.60 ^e^	97.83 ± 2.12 ^e^	43.80 ± 1.25 ^d^

All data are expressed as mean ± SD (n = 3). Means with different superscript letters within the same column are significantly different (*p* < 0.05).

**Table 3:** Droplet size, color parameters, and encapsulation rate of astaxanthin-loaded emulsions at different pH values.

pH	Droplet Size/nm	Encapsulation Rate/%	Color Parameters		
			L*	a*	b*
3	487.9 ± 4.5 ^b^	91.43 ± 0.26 ^d^	73.86 ± 0.01 ^d^	5.97 ± 0.01 ^b^	5.48 ± 0.01 ^a^
5	733.8 ± 3.9 ^e^	55.36 ± 1.55 ^a^	70.42 ± 0.01 ^a^	12.28 ± 0.01 ^e^	11.55 ± 0.01 ^e^
7	686.4 ± 2.7 ^c^	82.05 ± 1.54 ^b^	73.47 ± 0.01 ^d^	5.86 ± 0.01 ^a^	5.93 ± 0.01 ^b^
9	711.2 ± 0.7 ^d^	82.65 ± 0.63 ^b^	72.78 ± 0.02 ^c^	6.12 ± 0.01 ^c^	6.54 ± 0.01 ^c^
12	471.2 ± 8.3 ^a^	86.16 ± 0.25 ^c^	72.01 ± 0.02 ^b^	7.86 ± 0.01 ^d^	8.52 ± 0.01 ^d^

^a–e^ Different superscripts within the same column indicate significant differences between samples (*p* ≤ 0.05). The results were expressed as mean ± standard deviation (n = 3).

## Data Availability

The original contributions presented in this study are included in the article. Further inquiries can be directed to the corresponding author.

## Abbreviations

**Abbreviations**	**Full Name**
API	Almond protein isolate
AST	Astaxanthin
CI	Creaming index
CLSM	Confocal laser scanning microscopy
Cryo-SEM	Cryo-scanning electron microscopy
DI water	Deionized water
ESI	Emulsifying stability index
EAI	Emulsifying activity index
FC	Foaminess index
FS	Foam stability index
FT-IR	Fourier-transform infrared spectrometer
G′	Storage modulus
G″	Loss modulus
HCL	Hydrochloric acid
NaOH	Sodium hydroxide
OM	Optical microscope
PE	Pickering emulsion
SEM	Scanning electron microscope
SDS-PAGE	Sodium dodecyl sulfate-polyacrylamide gel electrophoresis
